# Pattern of gonadotropin secretion along the estrous cycle of C57BL/6 female mice

**DOI:** 10.14814/phy2.15460

**Published:** 2022-09-06

**Authors:** Daniela O. Gusmao, Henrique R. Vieira, Naira S. Mansano, Mariana R. Tavares, Ligia M. M. de Sousa, Frederick Wasinski, Renata Frazao, Jose Donato

**Affiliations:** ^1^ Department of Physiology and Biophysics Instituto de Ciencias Biomedicas, Universidade de Sao Paulo Sao Paulo Brazil; ^2^ Department of Anatomy Instituto de Ciencias Biomedicas, Universidade de Sao Paulo Sao Paulo Brazil

**Keywords:** follicle‐stimulating hormone, GnRH, HPG axis, Kisspeptins, luteinizing hormone

## Abstract

The pattern of gonadotropin secretion along the estrous cycle was elegantly described in rats. Less information exists about the pattern of gonadotropin secretion in gonad‐intact mice, particularly regarding the follicle‐stimulating hormone (FSH). Using serial blood collections from the tail‐tip of gonad‐intact C57BL/6 mice on the first day of cornification (transition from diestrus to estrus; hereafter called proestrus), we observed that the luteinizing hormone (LH) and FSH surge cannot be consistently detected since only one out of eight females (12%) showed increased LH levels. In contrast, a high percentage of mice (15 out of 21 animals; 71%) exhibited LH and FSH surges on the proestrus when a single serum sample was collected. Mice that exhibited LH and FSH surges on the proestrus showed c‐Fos expression in gonadotropin‐releasing hormone‐ (GnRH; 83.4% of co‐localization) and kisspeptin‐expressing neurons (42.3% of co‐localization) of the anteroventral periventricular nucleus (AVPV). Noteworthy, mice perfused on proestrus, but that failed to exhibit LH surge, showed a smaller, but significant expression of c‐Fos in GnRH (32.7%) and AVPV^Kisspeptin^ (14.0%) neurons. Finally, 96 serial blood samples were collected hourly in eight regular cycling C57BL/6 females to describe the pattern of LH and FSH secretion along the estrous cycle. Small elevations in LH and FSH levels were detected at the time expected for the LH surge. In summary, the present study improves our understanding of the pattern of gonadotropin secretion and the activation of central components of the hypothalamic–pituitary‐gonadal axis along the estrous cycle of C57BL/6 female mice.

## Graphical abstract

Our study exposed several challenges in working with gonad‐intact mice to evaluate the pattern of the HPG axis, especially during the LH surge. We observed that LH surge is often not observed in regular cycling C75BL/6 mice. We found evidence that in some cases in which a full LH surge is not observed, the HPG axis is somewhat activated, either through a small percentage of c‐Fos expression in MPO^GnRH^ and AVPV^Kisspeptin^ neurons or via a small peak in LH levels.

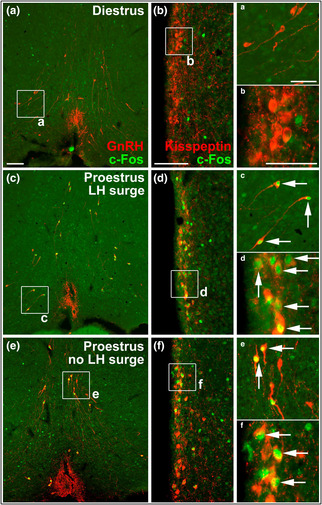

## 1 INTRODUCTION

The pituitary secretion of luteinizing hormone (LH) and follicle‐stimulating hormone (FSH) are regulated by hypophysiotropic gonadotropin‐releasing hormone (GnRH)‐expressing neurons located in the medial preoptic area (MPO) of the hypothalamus. Ovulation is triggered by a large burst of LH secretion, called the preovulatory LH surge. In rats, LH surge is observed in the transition between the light and dark phase of the proestrus day, which is frequently identified by an abundant number of epithelial cells in the vaginal smear and lasts approximately 10 h (Smith et al., 1975). FSH exhibits biphasic peaks at approximately the same time that the LH surge is detected (Smith et al., 1975). During the afternoon of proestrus, MPO^GnRH^ neurons exhibit c‐Fos expression, indicating that these neurons are activated immediately before the LH surge (Donato Jr. et al., 2009; Le et al., 1999; Lee et al., 1990). Of note, c‐Fos expression in MPO^GnRH^ neurons is not detected at any other time in the estrous cycle of female rats (Lee et al., 1990). c‐Fos expression immediately before the LH surge is also detected in the anteroventral periventricular and the rostral periventricular nuclei (here referred to as AVPV) (Le et al., 1999), and later studies have shown that this expression particularly occurs in kisspeptin‐expressing neurons (Christian & Moenter, 2010; Clarkson et al., 2008; Mohr et al., 2021; Robertson et al., 2009). Thus, the events that induce ovulation can be summarized as follows: AVPV^Kisspeptin^ neurons are activated by the progressive increase in estradiol levels along the estrous cycle; then, in the afternoon of proestrus, AVPV^Kisspeptin^ neurons strongly stimulate MPO^GnRH^ neurons, which in turn induce the LH surge (Christian & Moenter, 2010; Goodman et al., 2022; Herbison, 2020).

Much of the knowledge on the physiology of the hypothalamic–pituitary‐gonadal (HPG) axis of females was produced in rats, particularly regarding the LH surge. However, mice are becoming the dominant experimental model, among other reasons, due to the availability of a large number of genetically modified mouse models that allow elegant mechanistic and complex studies on the functioning of the HPG axis. Although we observe many aspects in common between rats and mice, some neuroendocrine differences may exist between these species (Campos et al., 2020; Campos et al., 2021; Wasinski et al., 2021). For example, the proestrus‐like cytology commonly found in the vaginal smears of rats is hardly observed in mice that “jump” from diestrus‐like cytology to an estrous‐like pattern (Bertolin & Murphy, 2014; Byers et al., 2012). Consequently, the first day of cornification in the vaginal smears of mice seems to be equivalent to the proestrus in rats. Thus, a better characterization of the HPG axis of gonad‐intact mice, including FSH determination, is necessary to allow a deeper understanding of this experimental model.

A key aspect that complicates neuroendocrine studies in mice, particularly those involving the secretion of pituitary hormones that show a complex and pulsatile secretion pattern, is the relatively low blood volume of these animals. However, remarkable progress in analyzing the pulsatile pattern of pituitary hormones has been achieved in mice (Czieselsky et al., 2016; Ongaro et al., 2021; Steyn et al., 2011). Thus, using serial blood collections from the tail tip, Czieselsky et al. (2016) demonstrated that, similar to rats, female mice also exhibit a surge‐like increase in LH levels at the transition from the light to dark phase on the first day of cornification (transition from diestrus to estrus; hereafter called proestrus). However, as reported by the authors, out of 11 mice on proestrus, only 7 exhibited increases in LH levels. Furthermore, the temporal and amplitude patterns were very different between the animals (Czieselsky et al., 2016). Despite this variability, several other studies using single or terminal blood collections confirmed that mice exhibit a LH surge at the transition from the light to dark phase on the proestrus (Bronson & Vom Saal, 1979; McQuillan et al., 2019; Ryan & Schwartz, 1980; Silveira et al., 2017; Wagenmaker & Moenter, 2017). On the other hand, the secretion pattern of FSH in the estrous cycle of mice has been less investigated. Thus, the present study aims to improve our knowledge about the secretion pattern of the gonadotropins along the estrous cycle of gonad‐intact C57BL/6 female mice. We performed several experiments using different approaches to determine circulating LH and FSH levels, especially at the moment expected for the LH surge. We also investigated the activation of the central components of the HPG axis during the LH surge.

## 2 MATERIAL AND METHODS

### 2.1 Animals

Twelve‐week‐old C57BL/6 female mice were maintained in standard conditions of light (12‐h light/dark cycle; lights on at 8:00) and temperature (22 ± 2°C). The animals were housed in groups of two to five mice per cage in ventilated racks and received a regular rodent chow (2.99 kcal/g; 9.4% kcal derived from fat; Nuvilab CR‐1, Quimtia) and filtered water ad libitum. The estrous cycle was monitored for approximately 20–25 consecutive days, and we used mice that showed regular estrous cyclicity. Females with regular estrous cyclicity needed to present at least three complete estrous cycles (clear switching between diestrus and cornification) within 20 days of analysis. The cycle phases were classified as follows: diestrus (mostly leucocytes, including metestrus) or cornification (mostly cornified or epithelial cells without distinction between proestrus and estrus). The animal procedures were previously approved by the Ethics Committee on the Use of Animals of the Institute of Biomedical Sciences at the University of São Paulo.

### 2.2 Serial blood collection in gonad‐intact and ovariectomized mice

A group of females was subjected to bilateral ovariectomy (*n* = 5), as previously described (Da Silva et al., 2014). The efficacy of the ovariectomy was assessed by weighing the uteri after euthanizing the animals. All ovariectomized (OVX) mice exhibited an absolute uterus mass lower than 20 mg. These animals were compared with gonad‐intact females on diestrus (*n* = 5). Before the blood collection, these mice were adapted 6 days per week to the procedure of tail‐tip blood sampling for 30 days. This procedure aimed to simulate the blood collection procedure and therefore minimize the effects of stress during the day of collection (Wasinski et al., 2020). After the adaptation period, the serial blood collection started at 13:00 (5 h after the lights were on). A surgical blade was used to remove a small portion of the tail tip (1 mm), allowing the collection of 5 μl blood with a 10 μl pipette at 30‐minute intervals for 4 h (9 serial collections in each mouse). After each blood collection, a fingertip pressure was gently applied to the tail tip to stop bleeding, and mice were allowed to move freely in their home cages with ad libitum access to food and water. The blood sample was immediately transferred to 105 μl (22× dilution) of phosphate‐buffered saline (PBS) containing 0.05% tween‐20 (PBS‐T), placed on dry ice, and stored at −80°C until analysis to determine LH and FSH levels.

### 2.3 Serial blood collection at the transition from the light to the dark phase to detect the LH surge

After the same period of adaptation, another group of regular cycling C57BL/6 female mice was used to possibly detect the LH surge. However, in this experiment, the serial blood collection at 30‐min intervals started 2 h before lights out and lasted until the first 2 h of the dark phase of the day. The mice were distributed into three groups according to their estrous cycle phase, which was determined by the analysis of the vaginal smears collected in the middle of the day: (1) mice in the diestrus on the day of collection and that continued on diestrus in the following day (*n* = 12), (2) mice on diestrus in the day of collection and that exhibited cornification in the following day (*n* = 8), and (3) mice that presented the first day of cornification (proestrus) in the day of collection (the day before they were on diestrus; *n* = 8). The 9 serial blood samples of each mouse were used to analyze LH and FSH levels. Photomicrographs of the vaginal smears were taken on the day before the collection, the collection day, and the following day.

### 2.4 Detection of the LH and FSH surges on proestrus using a single serum sample

To possibly detect the LH and FSH surges on proestrus using a single serum sample, mice were distributed into three experimental groups: gonad‐intact females on diestrus (*n* = 12), gonad‐intact females on proestrus (*n* = 21) and OVX mice (*n* = 10). This experiment was performed in two distinct animal cohorts and the hormone levels and the activation of central components of the HPG axis were analyzed separately in each cohort (Table 1 indicates the animals in each cohort). Mice were quickly anesthetized with isoflurane and blood was collected from the right atrium at the transition from the light to the dark phase at 20:30 ± 1 h (12‐h light/dark cycle; lights off at 20:00). A subgroup of these animals (cohort 2 shown in Table 1) was used to analyze the correlation between the hormone levels in samples obtained from tail‐tip (whole blood) from those collected in the right atrium (serum) before euthanasia. In these animals, the blood sample from the tail tip was collected and transferred to PBS‐T (20× dilution), followed by the collection of the blood from the right atrium, approximately 4 min later. Samples from the atrium were centrifuged to obtain the serum that was used to analyze LH and FSH levels (20× dilution in the assay). Immediately after the blood collection, a subgroup of mice was perfused with saline followed by a 10% buffered formalin solution (~150 ml/mouse). Brains and the uteri were carefully dissected. The uterus mass was determined, whereas the brains were submitted to a 30 min post‐fixation period in formalin at 4°C. Then, the brains were transferred to a 20% sucrose solution overnight. A freezing microtome (Leica SM2010R) was used to obtain 30 μm thick brain sections that were kept in cryopreservation buffer (20% glycerol, 30% ethylene glycol, and 50% PBS) at −20°C for posterior analyses. Each brain was divided into four brain series.

**TABLE 1:** Gonadotropin levels, uterus mass, and activation of central components of the HPG axis in mice perfused at the transition from the light to the dark phase.

Animal code/cohort	LH (ng/ml)	FSH (ng/ml)	Uterus mass (mg)	Uterus (mg/g b.w.)	% MPO^GnRH^ neurons expressing c‐Fos	% AVPV^kisspeptin^ neurons expressing c‐Fos
*Mice perfused on diestrus*						
#11/1	0.92	0.14	159.0	0.796	0.0%	0.0%
#12/1	0.48	0.84	129.1	0.569	0.0%	1.7%
#13/1	0.48	0.00	94.8	0.477	0.0%	0.0%
#14/1	0.66	0.86	64.8	0.313	0.0%	0.0%
#15/1	0.60	0.79	45.5	0.209	–	–
#16/1	0.24	1.86	41.3	0.199	0.0%	2.3%
#51/2	0.00	0.00	–	–	–	–
#52/2	0.08	0.00	–	–	–	–
#53/2	2.37	0.00	–	–	–	–
#54/2	0.58	0.00	–	–	–	–
#55/2	2.23	0.00	–	–	–	–
#56/2	0.32	0.00	–	–	–	–
**Mean**	**0.75**	**0.37**	**89.1**	**0.427**	**0.0%**	**0.8%**
*Mice perfused on proestrus and exhibiting LH surge*						
#20/1	28.84	7.28	149.6	0.749	84.1%	69.7%
#21/1	48.82	3.94	145.2	0.677	92.6%	25.0%
#22/1	64.72	7.74	136.8	0.613	83.8%	36.1%
#23/1	42.04	3.08	130.0	0.663	79.3%	19.0%
#24/1	44.90	4.22	123.2	0.595	67.5%	56.9%
#25/1	62.32	6.26	122.5	0.588	88.2%	24.3%
#26/1	52.92	9.34	119.6	0.573	88.5%	39.1%
#27/1	43.34	9.74	119.3	0.573	77.5%	43.0%
#28/1	46.26	10.78	118.2	0.583	75.6%	67.6%
#29/1	40.03	6.50	105.0	0.486	97.3%	–
#57/2	25.89	4.78	–	–	–	–
#58/2	13.72	6.60	–	–	–	–
#59/2	45.03	7.00	–	–	–	–
#60/2	51.08	6.07	–	–	–	–
#61/2	15.38	5.10	–	–	–	–
**Mean**	41.69****	6.56****	126.9*	0.610*	83.4%****	42.3%***
*Mice perfused on proestrus without exhibiting LH surge*						
#30/1	0.86	0.00	154.9	0.738	12.5%	7.7%
#31/1	0.08	9.44	72.8	0.345	0.0%	0.0%
#62/2	0.71	0.00	–	–	–	–
#63/2	4.75	1.88	–	–	44.4%	23.4%
#64/2	2.75	0.00	–	–	–	–
#65/2	6.27	0.00	–	–	74.1%	25.0%
**Mean**	**2.57####**	**1.89##**	**113.9**	**0.541**	32.7%* ^**##**^	14.0%* ^**#**^
*OVX mice*						
#41/1	4.78	36.56	16.1	0.069	–	–
#42/1	7.18	36.26	15.9	0.073	–	–
#43/1	6.74	36.40	14.0	0.062	–	–
#44/1	6.06	32.10	13.5	0.062	–	–
#45/1	9.80	41.74	12.6	0.063	–	–
#46/2	3.28	37.10	–	–	–	–
#47/2	6.27	38.39	–	–	–	–
#48/2	4.93	35.40	–	–	–	–
#49/2	8.13	39.74	–	–	–	–
#50/2	4.94	28.78	–	–	–	–
**Mean**	6.21**** ^**####**^	36.25**** ^**####**^	14.4** ^**####**^	0.066* ^**####**^	–	–

*Note*: –, not determined or tissue not available. *p < 0.05, **p < 0.01, ***p < 0.001, ****p < 0.0001 versus diestrus group; ^#^ *p* < 0.05, ^##^ *p* < 0.01, ^####^ *p* < 0.0001 versus LH surge group. These experiments were performed in two distinct cohorts of animals: cohort 1 (#11 to #45) and cohort 2 (#46 to #65). The hormone levels and activation of central components of the HPG axis were analyzed separately in each cohort.

### 2.5 Evaluation of the pattern of gonadotropin secretion along the estrous cycle of C57BL/6 female mice

To determine the pattern of gonadotropin secretion along the estrous cycle, 24 C57BL/6 female mice were acclimated to manipulation for 45 days. The estrous cycle was monitored daily for 25 days. Among 18 females that exhibited regular estrous cyclicity, 8 mice were selected for serial blood collection because all of them were in diestrus at the beginning of collections (*n* = 7) or on the second day of estrus (*n* = 1). Thus, we would have more chances to observe gonadotropin secretion during proestrus and consequently the occurrence of the LH surge. Blood samples were collected hourly during 4 consecutive days, totaling 96 serial collections for each animal. During each collection, the cage was gently removed from the ventilated rack and 4.4 μl of blood was collected from the tail tip and transferred to 105.6 μl of PBS‐T (25× dilution). Each blood collection took less than 1 min and the mice were returned to their home cage until the next collection1 h later. The animal facility room was equipped with red light to allow manipulation in the dark phase without interfering with the circadian rhythm. Six researchers took turns (12 h each) to carry out this experiment and all the researchers had previously participated in the adaptation of the animals. The vaginal cytology was obtained in the morning and in the evening of each day, immediately after blood collection (first and last hour of the light phase, respectively).

### 2.6 Hormone assessment

In‐house sensitive sandwich enzyme‐linked immunosorbent assays (ELISA) were adapted from published protocols to determine plasma LH (Brown et al., 2019; Quaresma et al., 2021) and FSH (De Paula et al., 2021; Ongaro et al., 2021) levels. A 96‐well high‐binding plate (9018, Corning) was coated with 50 μl of monoclonal mouse anti‐bovine LH beta subunit antibody (1:2500; Pablo Ross, UC Davis, 518B7; RRID: AB_2756886) or polyclonal guinea pig anti‐mouse FSH antibody (1:20,000; AFP1760191, NIDDK‐NHPP; RRID: AB_2665512) overnight at 4°C. After decanting the coating antibody, wells were incubated with 200 μl of blocking buffer (5% skim milk powder in PBS‐T) for 2 h at room temperature (RT). The standard curve consisted of a twofold serial dilution of mouse recombinant LH (AFP‐5306A, NIDDK‐NHPP) or rat recombinant FSH (rFSH‐RP‐2, AFP4621B, NIDDK‐NHPP) in 0.2% bovine serum albumin (BSA) PBS‐T from 0.00976 to 5 ng/ml and 0.03125 to 16 ng/ml, respectively. The wells were incubated with 50 μl of samples at 1:20, 1:22, or 1:25 dilutions, depending on the experiment, for 24 h at RT. The plate was washed in PBS‐T and the wells were incubated with 50 μl of polyclonal rabbit anti‐rat LH antibody (AFP240580Rb, NIDDK‐NHPP; RRID: AB_2665533) or rabbit anti‐rat FSH antibody (AFPC0972881, NIDDK‐NHPP; RRID: AB_2687903) diluted in blocking buffer at 1:40,000 and 1:6250, respectively, for 24 h at 4°C. After washing in PBS‐T, wells were incubated with 50 μl of horseradish peroxidase‐conjugated goat anti‐rabbit IgG antibody (A9169‐2 ml, Sigma‐Aldrich) diluted in 50% PBS, 50% blocking buffer at 1:30,000 for 90 min at RT. After a final wash in PBS‐T, wells were incubated with 100 μl of 2 mg/ml o‐phenylenediamine dihydrochloride (P1526, Sigma‐Aldrich) in citrate–phosphate buffer (pH 5.0) containing 0.02% hydrogen peroxide for 45 minutes at RT. The reaction was stopped with 50 μl of 3 M HCl. The absorbance was determined at 490 nm with a microplate reader (Epoch, Biotek) and the wavelength of 650 nm was used for background correction. The hormone levels in the samples were calculated by interpolating the optical density of the samples against a nonlinear regression of the standard curve. The LH and FSH assays have a lower limit of detection of 0.129 and 0.356 ng/ml, respectively, considering the dilution factors used. The intra‐assay coefficient of variation of the ELISA to detect LH and FSH was 4.43% and 6.32%, respectively. The inter‐assay coefficient of variation of the ELISA to detect LH and FSH was 7.51% and 18.65%, respectively.

### 2.7 Immunofluorescence staining

Brain sections containing the preoptic area were rinsed in 0.02 M potassium PBS, pH 7.4 (KPBS) buffer, and incubated for 1 h in a KPBS with 0.25% Triton X‐100 solution containing 3% of normal serum, followed by incubation in an anti‐c‐Fos antibody (1:10,000; Ab5, Millipore; RRID: AB_2314043) overnight under agitation at RT. Then, brain sections were rinsed in KPBS and incubated for 120 minutes in AlexaFluor^488^‐conjugated AffiniPure Fab Fragment donkey anti‐rabbit IgG (1:250; Jackson ImmunoResearch Laboratories, Cambridge, MA) at RT. After washing in KPBS, different brain series were either incubated in an anti‐GnRH (1:1000; ImmunoStar; RRID: AB_572248) or anti‐Kisspeptin 10 (1:2000; Millipore; RRID: AB_2296529) antibody overnight at RT. Subsequently, brain sections were rinsed in KPBS and incubated in AlexaFluor^594^‐conjugated whole IgG donkey anti‐rabbit (1:500; Jackson ImmunoResearch Laboratories) for 90 min. After washing in KPBS, brain sections were mounted onto gelatin‐coated slides and covered with Fluoromount G mounting medium (Electron Microscopic Sciences).

### 2.8 Image analysis

Epifluorescence or brightfield (from the vaginal smears) photomicrographs were acquired using a Zeiss Axioimager A1 microscope (Zeiss) using 10× (GnRH neurons and the vaginal smears) or 20× (Kisspeptin neurons) objectives. Images were analyzed with ZEN software (Zeiss, version 2.6) and Adobe Photoshop software (Adobe) was used for image arranging. The percentage of double‐labeled neurons was analyzed in approximately three rostral‐caudal levels of the medial preoptic area (MPO; Bregma between 0.345 and 0.545 mm) and the AVPV (Bregma between 0.345 and 0.02 mm). Only soma in sharp focus was considered for analysis, plotted electronically using the ImageJ software (http://rsb.info.nih.gov/ij/) and classified as either single‐ or double‐labeled. The Allen Brain Atlas (https://mouse.brain‐map.org/static/atlas) was used as a neuroanatomical reference.

### 2.9 Statistical analysis

The Prism software (GraphPad; RRID: SCR_002798) was used for the statistical analyses and generation of the graphs. Differences between the groups were analyzed by the unpaired Student's *t*‐test. Changes in gonadotropin levels along time were analyzed using repeated measures two‐way ANOVA. When the secretion pattern of gonadotropins was compared in different types of samples or in distinct moments of the estrous cycle, a paired two‐tailed Student's *t*‐test was used. Correlation analyses were determined using Pearson's correlation coefficient. We considered *p* values <0.05 to be statistically significant. The results are expressed as mean ± standard error of the mean.

## 3 RESULTS

### 3.1 OVX‐induced increases in gonadotropin levels can be detected using small volumes of serially collected blood from the tail tip

**FIGURE 1:**
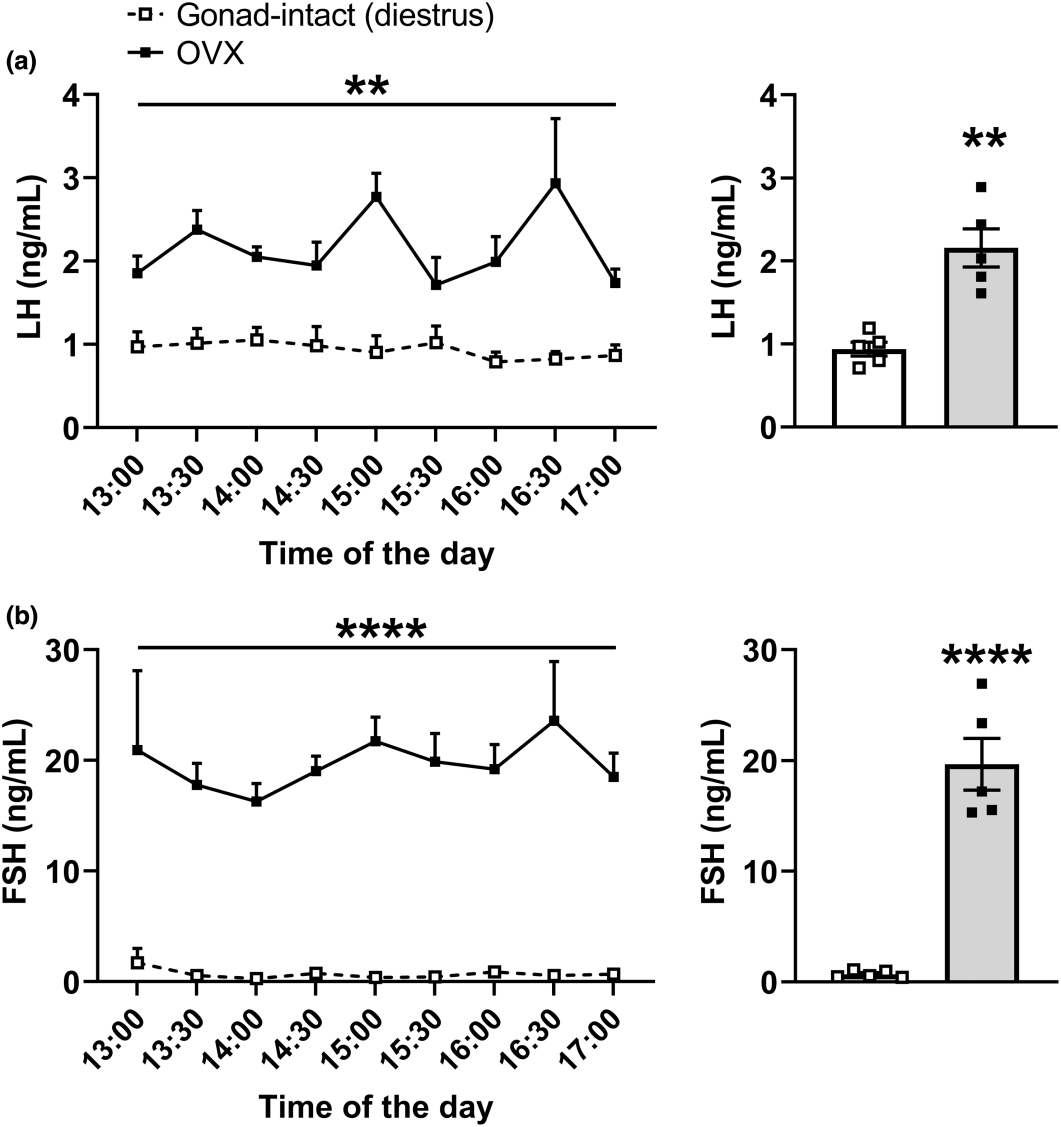
Circulating gonadotropin levels in gonad‐intact and OVX mice. (a, b) LH and FSH levels were determined in blood samples that were obtained from the tail tip at 30‐min intervals from C57BL/6 mice on diestrus (*n* = 5) and OVX mice (1 week after the surgery; *n* = 5) for 4 h. Blood collections started at 13:00 (12‐h light/dark cycle; lights on at 8:00). Bar graphs represent the average values. **P < 0.01; ****P < 0.0001.

Before evaluating the changes in LH and FSH levels in regular cycling females, we determined the sensitivity of our ELISA to detect physiological increases in gonadotropin levels using serial blood collections. For this purpose, 5 μl of blood samples were obtained from the tail tip at 30‐min intervals from gonad‐intact mice on diestrus and OVX mice for 4 h. As expected, OVX mice exhibited a significant increase in both LH (Figure 1a; *p* = 0.0011) and FSH (Figure 1b; *p* < 0.0001) levels, compared with gonad‐intact mice on diestrus. Thus, our ELISA can detect increases in gonadotropin levels using small volumes of serially collected blood from the tail tip.

### 3.2 Effects of sample type on LH and FSH values

**FIGURE 2:**
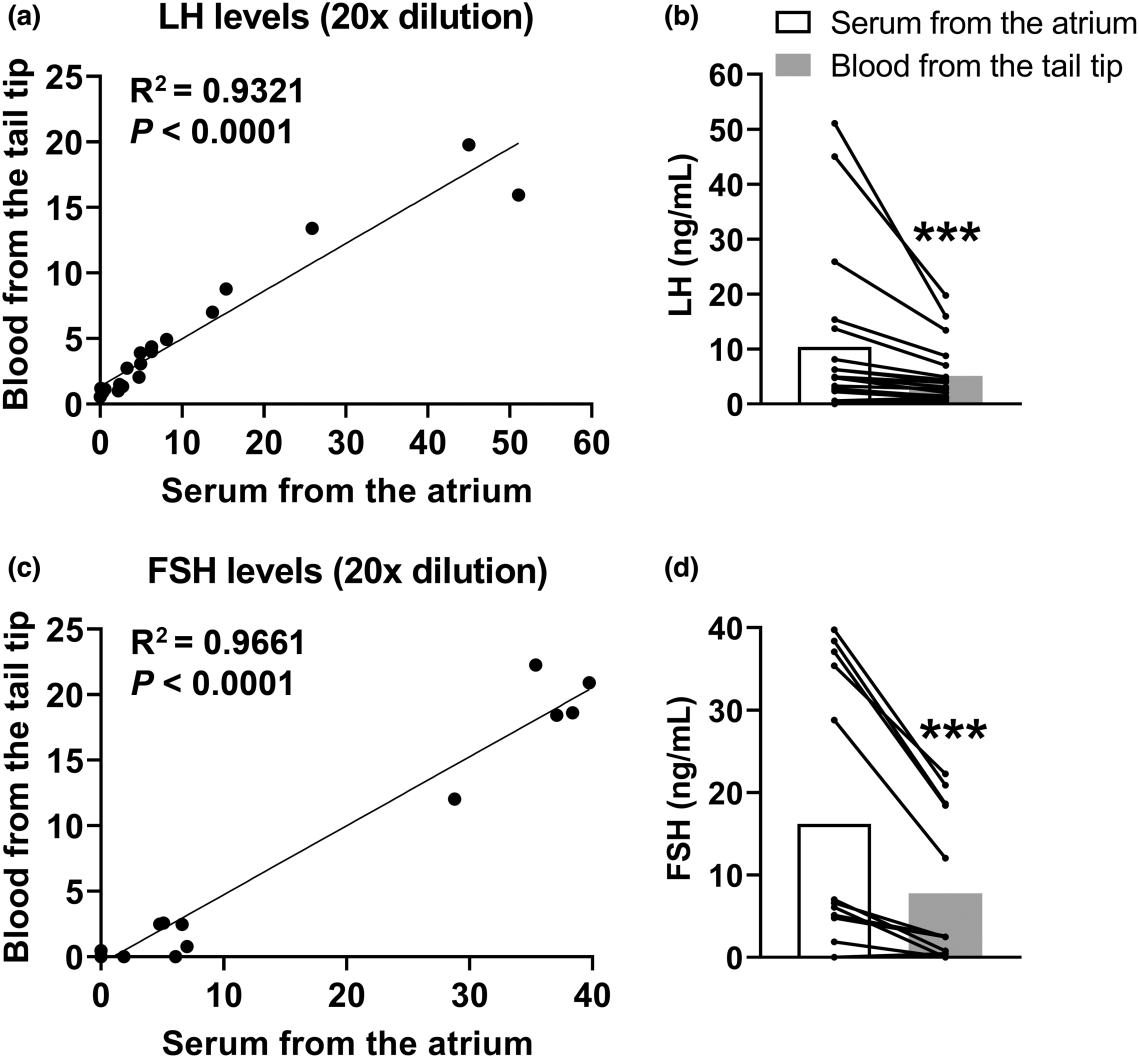
Effects of sample type on LH and FSH values. (a–d) correlation between the hormone levels in samples (*n* = 19) obtained from tail‐tip (whole blood) from those collected in the right atrium (serum) before euthanasia. These samples were collected with a time difference of approximately 4 min. ***P < 0.001.

Although serial collection of small volumes of whole blood from the tail tip represents a practical and well‐established method to analyze alterations in the levels of pituitary hormones along time (Chaves et al., 2022; Czieselsky et al., 2016; Dos Santos et al., 2022; Ongaro et al., 2021; Steyn et al., 2011; Wasinski et al., 2020), a decreased sensitivity has been reported for the FSH assay compared to serum samples (Ongaro et al., 2021). Thus, we compared the LH and FSH levels in samples obtained from tail‐tip (whole blood) from those collected in the right atrium (serum) before euthanasia. These samples were collected with a time difference of approximately 4 min. We used female mice in different situations: on diestrus (*n* = 6), OVX (*n* = 5) and on proestrus (*n* = 9). Using Pearson's coefficient, we observed a positive correlation between the hormone levels in samples from the tail tip (whole blood) or the atrium (serum), in both the LH (Figure 2a) and FSH (Figure 2c) assays. However, the absolute values were significantly decreased in the whole blood tail‐tip samples compared with serum samples from the atrium (Figure 2b,d). In this regard, the values obtained in the serum samples from the atrium were approximately twice as high as those obtained in the blood samples from the tail tip, either in the LH (Figure 2b) or FSH (Figure 2d) assays.

### 3.3 LH surge is not consistently detected on the first day of cornification in regular cycling C57BL/6 female mice

**FIGURE 3:**
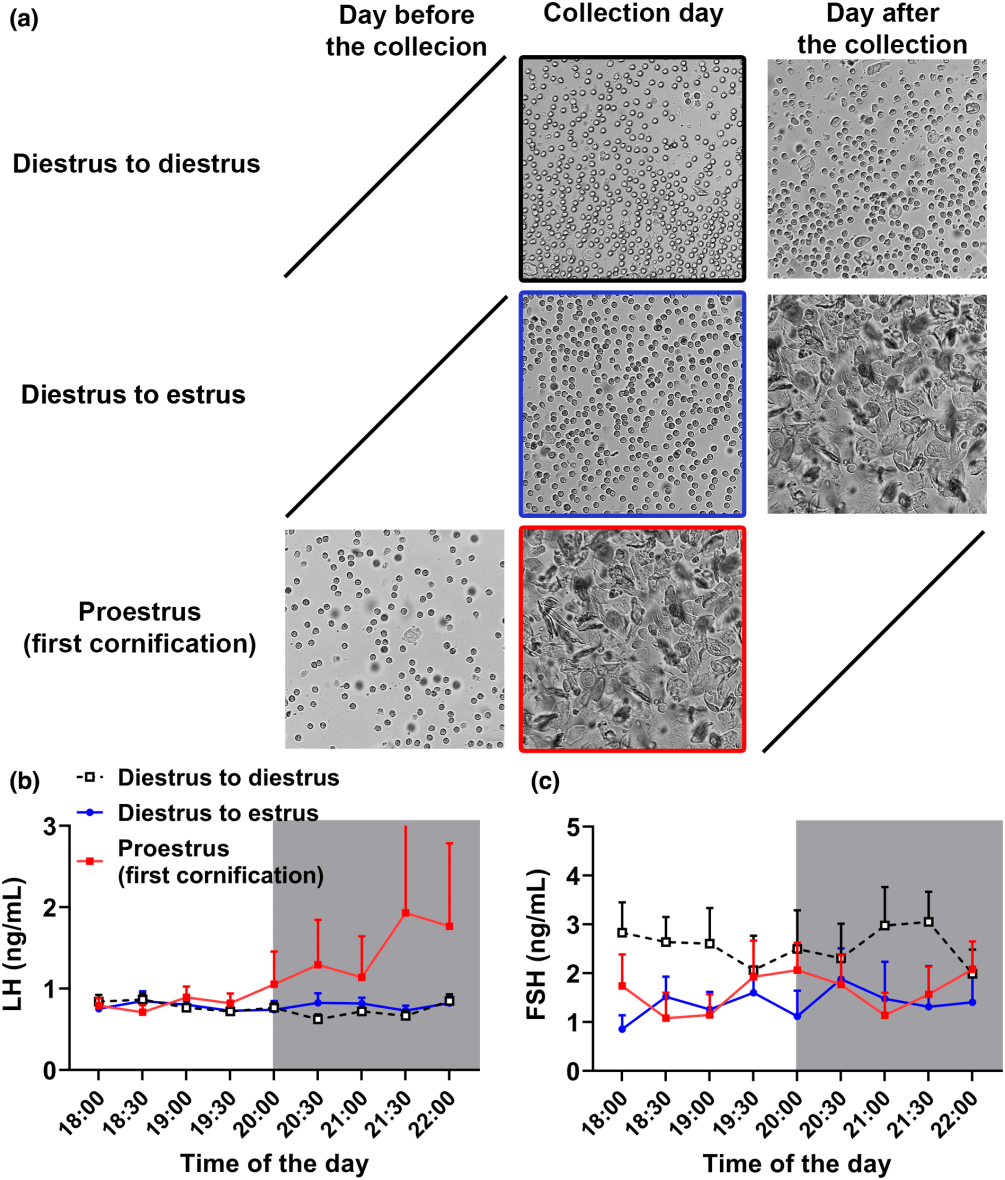
LH and FSH levels at the transition from the light to the dark phase. (a) Representative examples of the vaginal cytology of each group were provided in the figure (vaginal smears were collected in the middle of the day). (b, c) blood collections at 30‐min intervals started 2 h before lights out and lasted until the first 2 h of the dark phase of the day in mice on diestrus on the day of collection and that continued on diestrus on the following day (black line; *n* = 12), mice on diestrus in the day of collection and that exhibited cornification in the following day (blue line; *n* = 8) and mice that presented cornification in the day of collection (the day before they were on diestrus; red line; *n* = 8).

**FIGURE 4:**
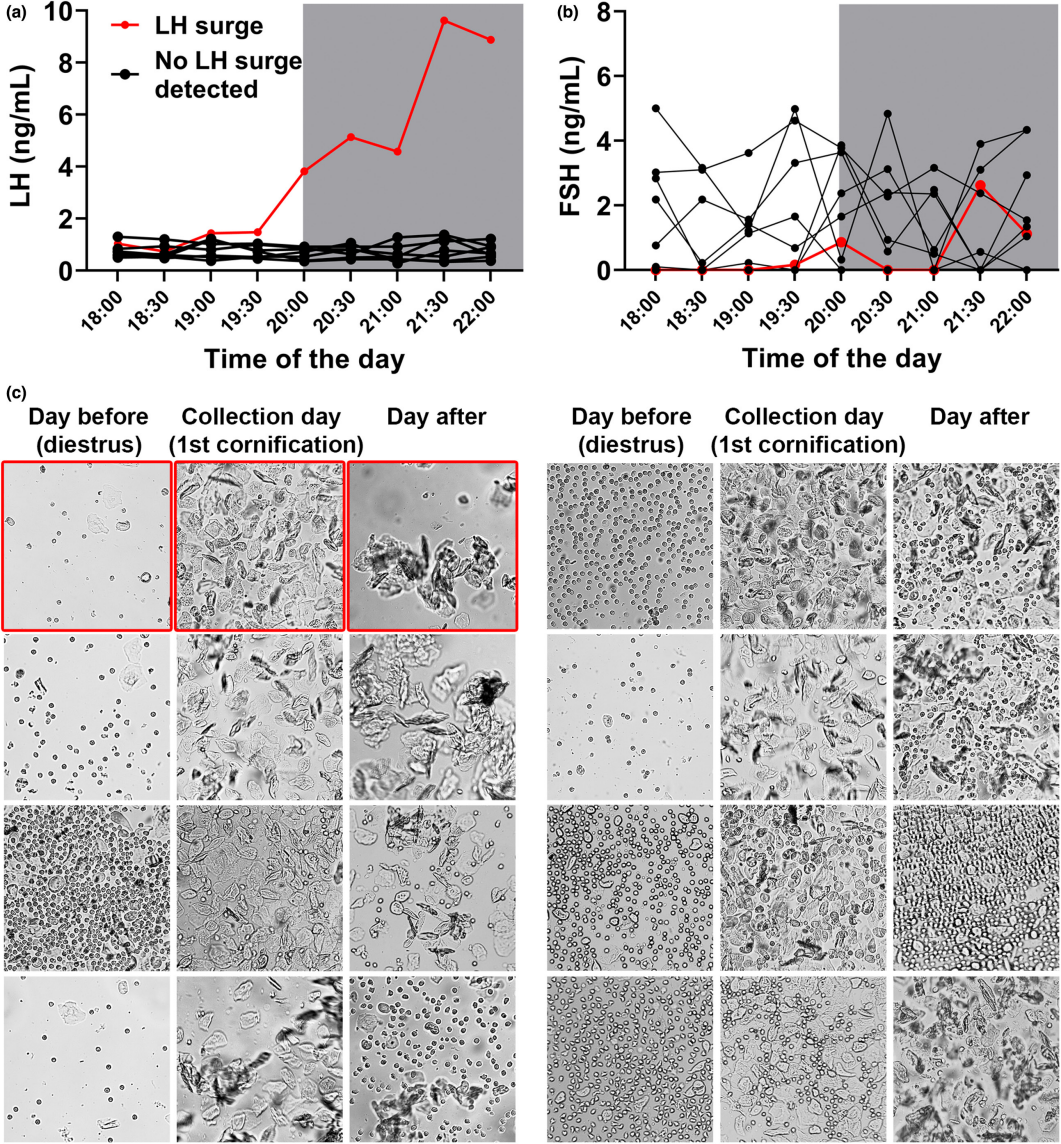
Individual information of mice in the first cornification/proestrus (transition from diestrus to estrus). (a, b) noticed that only one out of eight mice exhibited increased LH levels, which is in accordance with the LH surge (red line). (c) the vaginal cytology of the mice that exhibited LH surge was highlighted in red. The vaginal cytology of the seven mice that did not exhibit LH surge was also individually shown. The vaginal smears were collected in the middle of the day.

The same procedure of blood collection from the tail tip was now performed in C57BL/6 female mice that exhibited regular estrous cyclicity and were previously adapted to manipulation to detect the spontaneously occurring LH surge. Blood collections started 2 h before lights out and lasted until the first 2 h of the dark phase of the day, which is the time when LH surge is detected in rats and mice (Bronson & Vom Saal, 1979; Czieselsky et al., 2016; McQuillan et al., 2019; Ryan & Schwartz, 1980; Silveira et al., 2017; Smith et al., 1975; Wagenmaker & Moenter, 2017). The mice were distributed into three groups according to their estrous cycle phase. Representative examples of vaginal smears are shown in Figure 3a. No statistical differences were observed in LH (Figure 3b) and FSH (Figure 3c) levels in mice on proestrus, compared with mice that had their blood collected on the afternoon of diestrus, regardless of whether they remained in diestrus or had cornification in the following day. When gonadotropin levels of the first cornification group were examined individually (*n* = 8; the vaginal cytology of all mice was individually shown in Figure 4c), we noticed that only one female exhibited an increase in LH levels (Figure 4a), resembling the pattern expected of the LH surge (Czieselsky et al., 2016). The LH levels of other mice that also presented cornification remained unaltered during the collection period. FSH levels were not increased in the mouse that exhibited LH surge, as compared with the remaining mice on the first day of cornification (Figure 4b).

### 3.4 A high percentage of C57BL/6 females exhibits LH and FSH surge when using a single serum sample collected on the proestrus

Given the very low percentage of mice exhibiting LH surge in our previous experiment and the relatively limited information regarding the pattern of LH surge in gonad‐intact mice, the following experiment was designed to simultaneously determine whether the activation of key neuronal populations of the HPG axis reflects in increases in gonadotropin levels at the time of the LH surge. Thus, blood samples were collected in two separate cohorts of regular cycling C57BL/6 females on the transition from the light to the dark phase to analyze LH and FSH levels. All mice perfused on diestrus showed low LH levels, indicating the absence of the LH surge (Table 1). In contrast, 15 out of 21 mice (71%) on the proestrus exhibited very high LH levels, suggesting that they were at the time of LH surge (Table 1). As a comparison, a group of OVX mice was also analyzed and LH levels of mice at the LH surge were almost 7 times higher compared with OVX animals (Table 1). FSH levels were also significantly increased at the time of LH surge, as compared with mice on diestrus (Table 1). However, in contrast to that observed in LH levels, FSH serum concentration of OVX mice was higher than the levels observed in mice during the LH surge (Table 1). Regarding the mice perfused on proestrus but that did not show LH surge, one of them (#31) had high FSH levels despite a very low LH concentration (Table 1). Mice that exhibited LH surge had the uterus mass significantly increased, as compared with mice on diestrus (Table 1). Taken together, a high percentage of C57BL/6 females exhibited LH surge when using a single serum sample collected on the proestrus. Furthermore, FSH levels are also increased at the time of the LH surge.

### 3.5 c‐Fos expression in MPO^GnRH^ and AVPV^Kisspeptin^ neurons during the LH surge in mice

**FIGURE 5:**
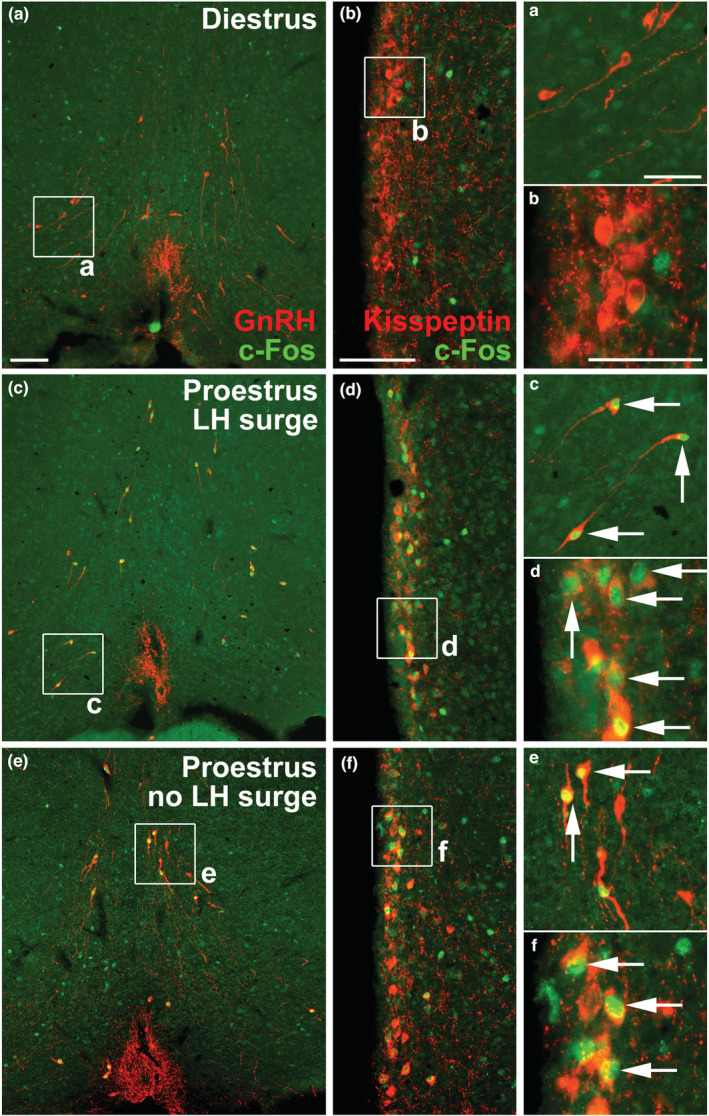
Activation of MPO^GnRH^ and AVPV^Kisspeptin^ neurons at the LH surge in regular cycling C57BL/6 female mice. (a, b), MPO^GnRH^ and AVPV^Kisspeptin^ neurons (red staining) do not exhibit c‐Fos expression (green staining) in mice on diestrus. Perfusion occurred during the transition from the light to the dark phase (20:30 ± 1 h; 12‐h light/dark cycle; lights off at 20:00). (c, d) robust c‐Fos expression in MPO^GnRH^ and AVPV^Kisspeptin^ neurons in mice that exhibited the LH surge. These mice show cornification on the perfusion day (transition from diestrus to estrus) and they had high LH levels during the perfusion (see Table 1). Arrows indicate examples of double‐labeled neurons. E‐F, mice perfused on proestrus but that did not have LH surge showed co‐localization of c‐Fos in MPO^GnRH^ and AVPV^Kisspeptin^ neurons (see Table 1). Scale bars = 100 μm (50 μm in the high magnification insets).

MPO^GnRH^ and AVPV^Kisspeptin^ neurons exhibit c‐Fos expression at the time of LH surge in rats (Donato Jr. et al., 2009; Le et al., 1999; Lee et al., 1990). Thus, the co‐localization between c‐Fos and GnRH or Kisspeptin was analyzed in a subset of the mice euthanized on the transition from the light to the dark phase. No MPO^GnRH^ neuron and only a very small percentage of AVPV^Kisspeptin^ neurons exhibited c‐Fos expression in mice perfused on diestrus (Table 1 and Figure 5a,b). In contrast, all mice that exhibited high LH levels on proestrus, indicating the occurrence of LH surge (Table 1), showed a very high percentage of MPO^GnRH^ neurons exhibiting c‐Fos (83.4%; Table 1 and Figure 5c). c‐Fos expression was also consistently observed in AVPV^Kisspeptin^ neurons of mice that exhibited LH surge, although the percentage of co‐localization was less (42.3%) than that observed in MPO^GnRH^ neurons (Table 1 and Figure 5d). No differences in the number of GnRH neurons were observed between mice perfused on diestrus (15.7 ± 1.9 cells/section) or on proestrus (14.3 ± 1.0 cells/section; *p* = 0.4679). In contrast, the number of kisspeptin neurons was increased in the group of mice that exhibited LH surge (24.9 ± 1.0 cells/section), compared with mice on diestrus (20.1 ± 0.6 cells/section; *p* = 0.0035). Noteworthy, three mice perfused on proestrus but that exhibited low LH levels (Table 1; mice #30, #63, and #65) showed c‐Fos expression in MPO^GnRH^ and AVPV^Kisspeptin^ neurons, although in a lower percentage than that observed in mice that had the LH surge (Table 1 and Figure 5e,f). No statistically significant correlation between LH levels, FSH levels, uterus mass, % of MPO^GnRH^ neurons expressing c‐Fos, and % of AVPV^Kisspeptin^ neurons expressing c‐Fos was observed in the mice that exhibited LH surge.

### 3.6 Gonadotropin secretion pattern along the estrous cycle of C57BL/6 female mice

**FIGURE 6:**
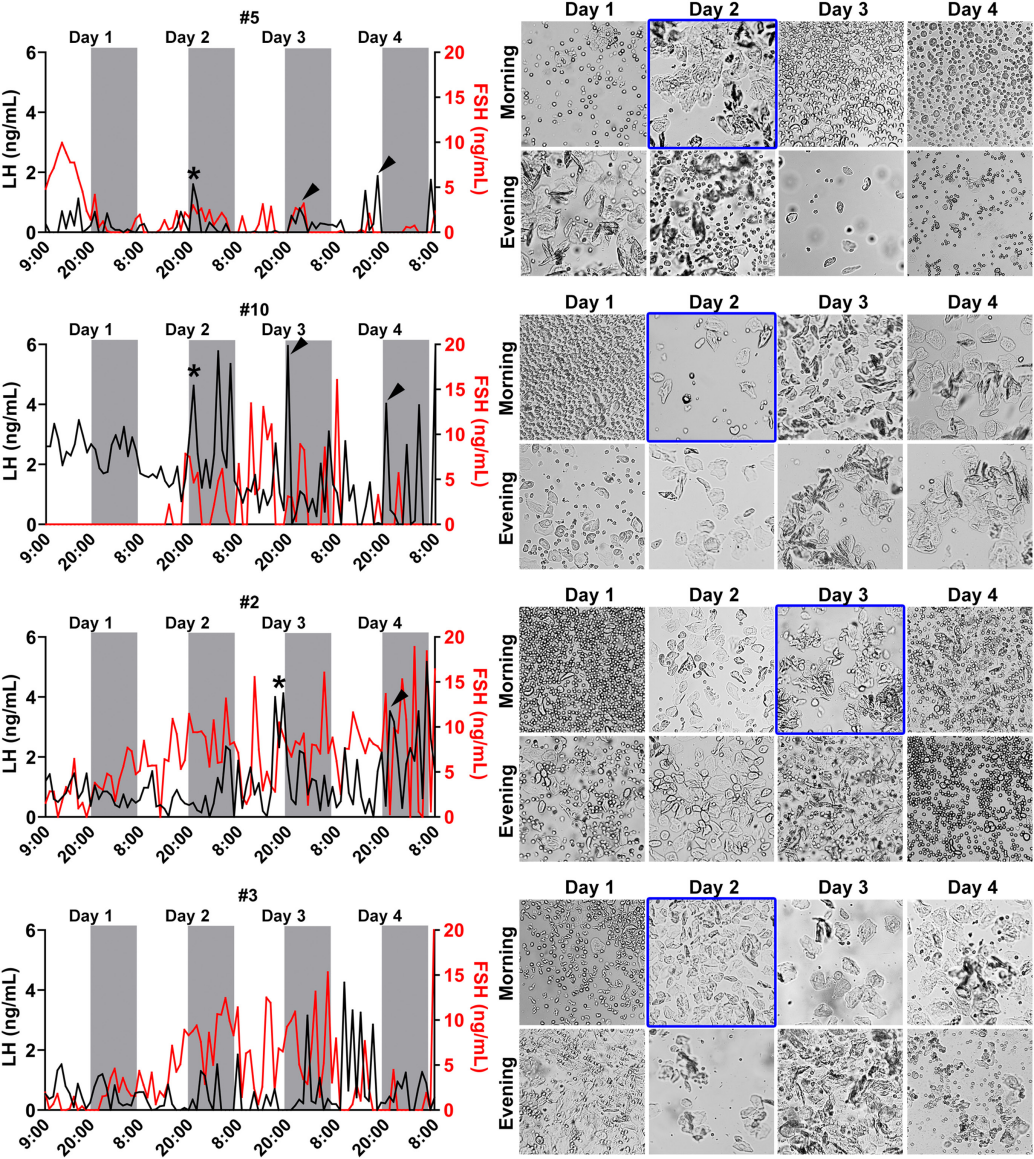
The pattern of LH and FSH secretion throughout the estrous cycle of C57BL/6 mice. LH (black line) and FSH (red line) levels were determined hourly through blood collections obtained from the tail tip. The estrous cycle was determined in the morning (in the first hour of the light phase) and in the evening (in the last hour of the light phase) during 4 consecutive days (12‐h light/dark cycle; lights on at 8:00). The four mice described in the figure presented transition from diestrus to estrus during the experiment, which was highlighted in blue. The asterisks indicate potential elevations in LH levels at the time expected for the LH surge (transition from the light to the dark phase on the first day of cornification). The arrowheads indicate similar LH peaks in the following days.

**FIGURE 7:**
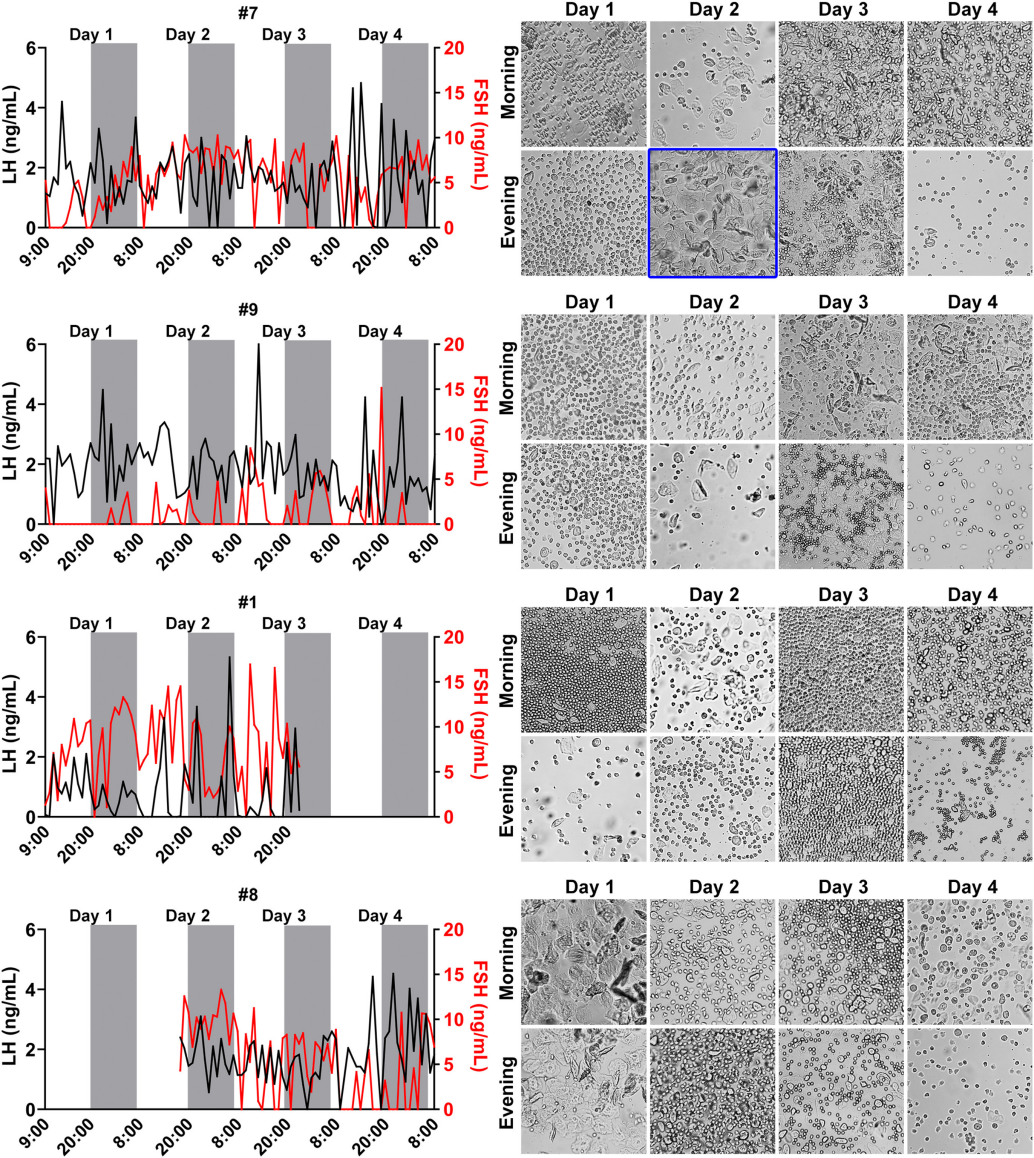
The pattern of LH and FSH secretion along the estrous cycle in mice. LH (black line) and FSH (red line) levels were determined hourly through blood collections obtained from the tail tip. The estrous cycle was determined in the morning (in the first hour of the light phase) and in the evening (in the last hour of the light phase) during 4 consecutive days (12‐h light/dark cycle; lights on at 8:00).

In the next experiment, blood samples were collected hourly in eight regular cycling C57BL/6 females during 4 consecutive days. The LH and FSH levels in each hour, as well as the vaginal cytology obtained in the morning and the evening of each day were shown individually (Figures 6 and 7). Five out of eight mice exhibited a transition from diestrus to estrus during the analyzed period (Figures 6 and 7; the cornification was highlighted in blue in the pictures of the vaginal cytology). In none of these cases, we observed increases in LH levels resembling the pattern of the LH surge. However, a peak in LH levels was observed approximately at the transition from the light to the dark phase in three mice (#5, #10, and #2; the LH peaks were indicated by asterisks; Figure 6). Interestingly, these mice also exhibited similar LH peaks nearby the beginning of the dark phases in the days following the cornification (these subsequent peaks are indicated by arrowheads in Figure 6). FSH levels showed a very irregular secretion pattern along the estrous cycle, without a noticeable increase at the time expected for the LH surge. Three mice (#9, #1 and #8) did not exhibit a transition from diestrus to estrus during the collection period, as observed in the vaginal cytology (Figure 7).

**FIGURE 8:**
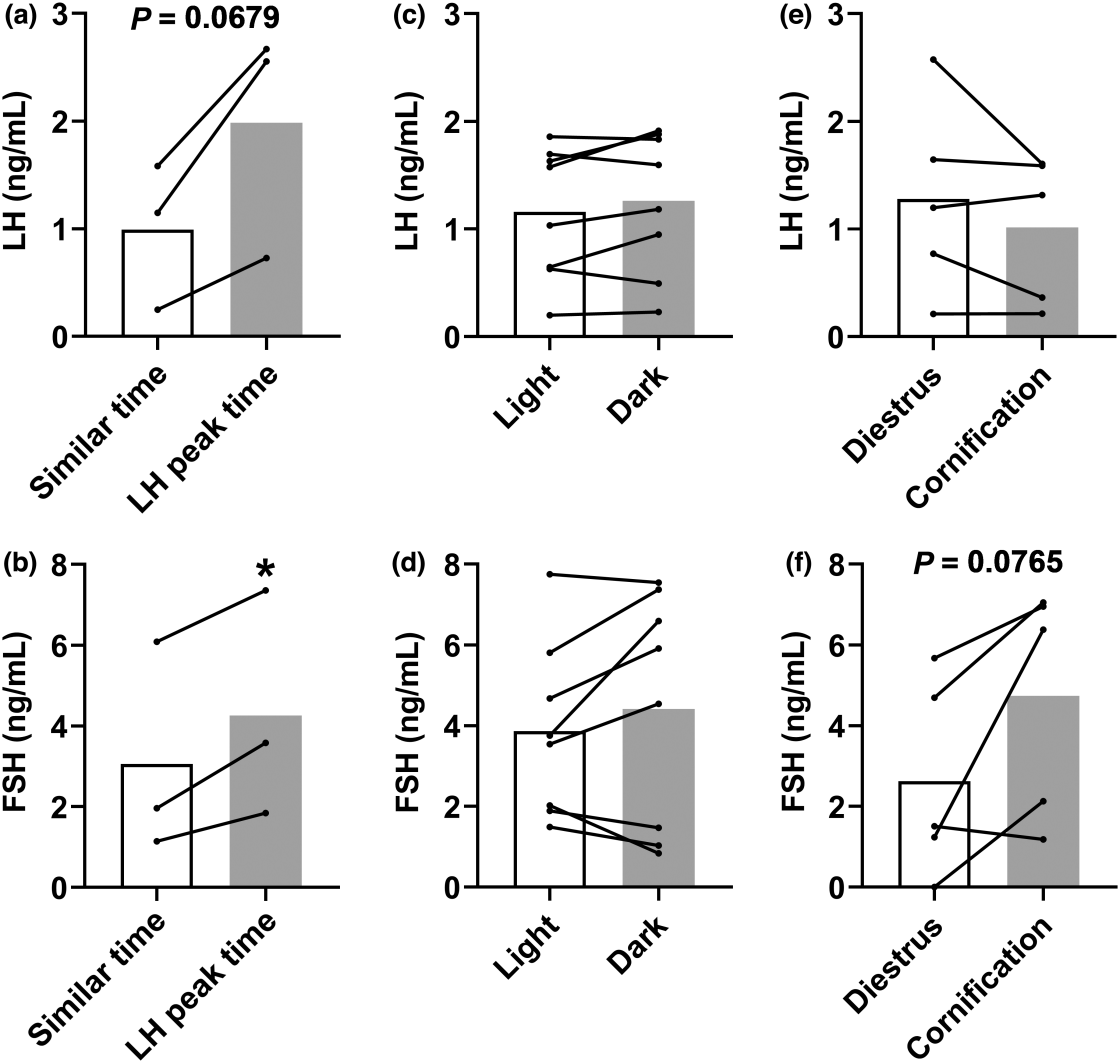
Analysis of the pattern of LH and FSH secretion throughout the estrous cycle of C57BL/6 mice. (a, b) differences in mean LH and FSH levels at the time the LH peak was detected, in comparison with similar times in the remaining days of the estrous cycle. Data from mice #2, #5, and #10. (c, d) differences in mean LH and FSH levels between the light and dark phase of the day. Data from mice #1, #2, #3, #5, #7, #8, #9, and #10. (e, f) differences in mean LH and FSH levels between the diestrus and the cornification days (including proestrus and estrus). Data from mice #2, #3, #5, #7, and #10. *P < 0.05.

We compared the LH and FSH levels at the time we observed the LH peak of mice #5, #10, and #2 on the first day of cornification, with the gonadotropin levels observed at the same time of the other analyzed days (Figure 8a,b). Although no LH surge was observed in these mice, LH levels tended to be higher (*p* = 0.0679) on the day of the first cornification (peaks indicated by asterisks in Figure 6), compared with the LH levels observed on the other days analyzed, considering the same time of the day (Figure 8a). Regarding FSH, a significant increase (*p* < 0.05) was observed at the LH peak time, as compared with the other days (Figure 8b). Next, mean LH and FSH levels were compared between the light and dark phases of the day in the eight animals evaluated. No differences in the LH and FSH levels were observed between the light and dark phases of the day (Figure 8c,d). Finally, mean LH levels were similar between the diestrus days and the cornification days, including proestrus and estrus (Figure 8e). However, FSH levels tended to be higher (*p* = 0.0765) on the days of cornification, compared with the days on diestrus (Figure 8f).

## 4 DISCUSSION

The present study aimed to describe the pattern of gonadotropin secretion and the activation of central components of the HPG in gonad‐intact C57BL/6 female mice, particularly during the LH surge. After confirming the capacity of our protocol to detect physiological increases in LH and FSH levels through serial blood collections from the tail tip using OVX mice, we investigated the changes in these hormones at the time expected for the LH surge. Surprisingly, only 1 out of 8 mice (12%) exhibited LH surge on the proestrus. Previous studies have already described the occurrence of LH surges in mice (Bronson & Vom Saal, 1979; Czieselsky et al., 2016; Ryan & Schwartz, 1980). However, two studies used male exposition to induce or increase the changes of LH surge (Bronson & Vom Saal, 1979; Ryan & Schwartz, 1980), whereas in another study that did not expose the female mice to males, the authors observed that 63% of females exhibited LH surge in the proestrus day (Czieselsky et al., 2016). Additionally, the pattern of LH surge was very variable between the mice (Czieselsky et al., 2016). Noteworthy, the pattern of the ovulatory LH surge is also extremely variable in women (Park et al., 2007). Taken together, a regular estrous cyclicity and a clear confirmation of the first cornification in the vaginal smears are not a guarantee that the LH surge can be observed in mice.

Some aspects may have contributed to the low percentage of mice exhibiting LH surge in our experiment. First, our mice are exclusively housed in ventilated racks equipped with HEPA filters, so the female's exposure to male cues, like pheromones, is minimal. Thus, although mice do not require copulation or male exposure to trigger the ovulatory LH surge, previous studies indicate that male exposure can facilitate this event (Ryan & Schwartz, 1980). Accordingly, a former study that observed LH surge in male‐exposed female mice on proestrus did not find increased LH levels in the absence of male exposure (Ryan & Schwartz, 1980). Additionally, mating increases the percentage of MPO^GnRH^ neurons expressing c‐Fos in OVX mice treated with estradiol to induce the LH surge (Wu et al., 1992). Therefore, some level of male exposure should be considered in experiments wishing to study LH surge in gonad‐intact mice. The second factor that may be taken into consideration is the stress involved in serial blood collections. Although all mice were extensively adapted to the procedure of tail‐tip blood collection, we cannot discard that repeated blood sampling may have blocked the induction of the LH surge. Although chronic or acute stress does not disrupt the estrous cyclicity in female mice, 5 h of exposure to psychosocial stress on midmorning of proestrus blunted the LH surge in 14 out of 24 mice on proestrus (58%), while the remaining mice (10 out of 24; 42%) still exhibited LH surge with similar amplitude to nonstress controls (Wagenmaker & Moenter, 2017). Considering that the blood collection in our study started 2 h before the beginning of the LH surge and the stress induced by this procedure is likely significantly lower than the protocol previously tested (Wagenmaker & Moenter, 2017), we believe that the low occurrence of LH surge in our experiment cannot be explained by the blood collection procedure. However, it is desired that future studies may determine glucocorticoid levels in experiments involving serial blood collections to measure LH surge. Finally, the sensitivity of our assays to detect changes in LH and FSH levels from the whole blood collected from the tail‐tip is probably lower than that obtained from serum/plasma samples. In the paper that described the development of a highly sensitive ELISA to measure circulating FSH levels in mice, the authors reported decreased sensitivity of the assay to detect variations in FSH levels using tail‐tip blood samples (Ongaro et al., 2021). Therefore, the lack of a significant increase in FSH levels in the mouse that exhibited the LH surge may be related to this lower sensitivity of the assay in detecting FSH from tail‐tip blood samples. This limitation has not been reported for the LH assay (Czieselsky et al., 2016). In the current study, we observed a strong correlation between the hormone levels in samples from the tail tip (whole blood) or the atrium (serum). However, the absolute values were significantly lower in the whole blood tail‐tip samples compared with serum samples from the atrium, even though we used the same dilution (20×) in the ELISA. This result explains the differences in LH levels when the LH surge were analyzed by serial blood collections from the tail tip or a single collection from the atrium. In addition, these lower values in serial collections can make it difficult to identify the pre‐ovulatory peaks.

Classical studies using female rats or ewes have shown that the ovulatory LH surge is preceded by the activation of MPO^GnRH^ and AVPV^Kisspeptin^ neurons (Hoffman et al., 2011; Le et al., 1999; Lee et al., 1990; Merkley et al., 2012; Moenter et al., 1993; Smith et al., 2009). Although the c‐Fos expression in MPO^GnRH^ and AVPV^Kisspeptin^ neurons has been described in female mice, in the majority of the studies, LH surge was induced by sex steroid treatment in OVX mice (Clarkson et al., 2008; Dungan et al., 2007; Robertson et al., 2009; Wu et al., 1992). Thus, the activation pattern of MPO^GnRH^ and AVPV^Kisspeptin^ neurons during the LH surge of gonad‐intact mice has been less described. Here, we showed that a high percentage (>80%) of MPO^GnRH^ neurons located around the organum vasculosum lamina terminalis exhibit c‐Fos at the LH surge. This percentage is higher than that observed in female rats on proestrus or in estradiol‐treated ewes (approximately 40%) (Donato Jr. et al., 2009; Lee et al., 1990; Moenter et al., 1993). To our knowledge, only Zhang et al. (2014) assessed c‐Fos expression in MPO^GnRH^ neurons in gonad‐intact mice on proestrus and they observed 66% of co‐localization, which is in accordance with our data demonstrating a higher percentage of MPO^GnRH^ neurons expressing c‐Fos in mice, as compared with other species. It is well‐known that AVPV^Kisspeptin^ neurons are required for estrogen positive feedback (Christian & Moenter, 2010; Clarkson et al., 2008; Mohr et al., 2021; Robertson et al., 2009). We found that approximately 42% of AVPV^Kisspeptin^ neurons exhibit c‐Fos during the LH surge in gonad‐intact mice. Another study observed that 25% of AVPV^Kisspeptin^ neurons exhibited c‐Fos in regular cycling mice on proestrus (Zhang et al., 2014). Noteworthy, while the percentage of MPO^GnRH^ neurons exhibiting c‐Fos at the LH surge was consistently high in all mice (range: 67.5%–97.3%), a greater variability was observed in the percentage of AVPV^Kisspeptin^ neurons exhibiting c‐Fos (range: 19.0%–69.7%). The reasons for this variability are unknown since no significant correlation was observed between c‐Fos expression with serum LH and FSH levels or uterus mass. As observed in rats or ewes (Le et al., 1999; Lee et al., 1990; Moenter et al., 1993), virtually no c‐Fos expression was observed in MPO^GnRH^ and AVPV^Kisspeptin^ neurons of mice on diestrus. Noteworthy, from four mice that were perfused on the proestrus but showed low LH levels, mice #30, #63, and #65 exhibited evident expression of c‐Fos in MPO^GnRH^ and AVPV^Kisspeptin^ neurons, even though in a lower percentage than that observed in mice that had the LH surge. Thus, it is possible that the estrogen positive feedback did not reach a threshold level sufficient to induce the LH surge in these animals. More studies are necessary to determine whether this is a common phenomenon in female mice, possibly explaining the frequent absence of LH surges in regular cycling mice, as observed in our study.

In our last experiment, blood samples were collected hourly in eight regular cycling C57BL/6 females during 4 consecutive days to determine LH and FSH levels. Five females exhibited a transition from diestrus to estrus along the collection period, but none of them had a surge‐like increase in LH and FSH levels at the expected time for the LH surge. However, a small peak in LH levels was observed in three mice (#5, #10 and #2) close to the transition to the dark phase of the day. When LH and FSH levels were analyzed during this period and compared with the levels found at the same time of the other days, we could observe a tendency toward an increase in LH and FSH levels. However, similar increases in LH levels were observed in other moments of the estrous cycle, which makes it difficult to clearly determine whether we detected only random variations of the secretion of gonadotropins or the expected surges in the afternoon of proestrus. Interestingly, similar elevations in LH levels were observed at the transition from the light to dark phase in the days following proestrus. Although speculative, it is possible that the mechanisms that induce the LH surge in these animals reached a subthreshold level. Thus, the failure in presenting a “full LH surge” and possibly ovulation may have triggered additional attempts in the following days. Estradiol treatment in OVX rats can induce successive LH surges (Caligaris et al., 1971; Legan et al., 1975; Legan & Karsch, 1975). Whether the successive LH peaks observed in our study have any similarity to the mechanisms associated with the repeated estradiol‐induced LH peaks in OVX rats is unknown.

Estradiol‐induced LH surge in OVX mice exhibits a lower amplitude than the levels reached in gonad‐intact female mice on proestrus (Czieselsky et al., 2016; Silveira et al., 2017). Interestingly, mean GnRH neuron firing rate was similarly increased in proestrus and OVX + estradiol mice, although some differences in the pattern of action potentials were observed between these conditions (Silveira et al., 2017). This study provides evidence that differences in the activation pattern of the central components of the HPG axis may produce LH surges with different amplitudes (Silveira et al., 2017). Therefore, it would be interesting that the activity of MPO^GnRH^ and AVPV^Kisspeptin^ neurons may be compared between gonad‐intact female mice that exhibited a “full LH surge” with those that showed smaller peaks.

Taking advantage of the continuous blood collection throughout the day, we were able to compare the mean LH and FSH levels between the dark and light phases of the day. Interestingly, we did not observe significant circadian differences in the mean levels of these hormones. It is worth mentioning that our experiment was not designed to measure LH (and FSH) pulses. Such an experiment would require more frequent blood collections (e.g., 5‐ to 10‐min intervals) (Czieselsky et al., 2016) than the hourly assessment performed here. We also compared mean LH and FSH levels in different phases of the estrous cycle. In accordance with a previous study that assessed LH levels at 10 min intervals for 2 h (Czieselsky et al., 2016), we observed similar mean LH levels between the diestrus (including metestrus) and cornification (both proestrus and estrus). However, a tendency toward an increase in mean FSH levels was observed in mice during cornification, as compared with diestrus. Although no peak in FSH levels was observed on the first day of cornification (proestrus), our findings are in accordance with data in rats that show higher FSH levels in the proestrus and on the first day of estrus (Smith et al., 1975).

In summary, our study exposed several challenges in working with gonad‐intact mice to evaluate the pattern of the HPG axis, especially during the LH surge. We observed that LH surge is often not observed in regular cycling C75BL/6 mice. As a result of this difficulty, many researchers prefer to study OVX females treated with estrogen to investigate aspects related to the LH surge. At least using a single blood collection, we confirmed that FSH levels are also increased at the time of the LH surge (Ongaro et al., 2021). In addition, we found a higher percentage of MPO^GnRH^ neurons expressing c‐Fos during the LH surge in mice, as compared with other species. Conversely, the percentage of AVPV^Kisspeptin^ neurons expressing c‐Fos during the LH surge in mice is compatible with the values found in rats. Finally, we found evidence that in some cases in which a full LH surge is not observed, the HPG axis is somewhat activated, either through a small percentage of c‐Fos expression in MPO^GnRH^ and AVPV^Kisspeptin^ neurons or via a small peak in LH levels. Thus, it is possible that sometimes gonad‐intact mice may present a subliminal activation of the HPG at the time expected for the LH surge. To our best knowledge, this phenomenon has not been described in rats and may help to explain the irregular occurrence of LH surges in gonad‐intact mice. Taken together, the findings described in our study will help future studies aiming to investigate the HPG axis and the LH surge in gonad‐intact mice.

## AUTHORS' CONTRIBUTION

**Daniela O Gusmao** did formal analysis and investigation. Henrique R Vieira, Naira S Mansano, Mariana R Tavares, Ligia MM de Sousa, and Frederick Wasinski did investigation. **Renata Frazao** contributed to conceptualization, methodology, and funding acquisition. **Jose Donato Jr** contributed to conceptualization, data Curation, writing –original draft, project administration, and funding acquisition.

## CONFLICT OF INTEREST

The authors declare no conflicts of interest.

## Data Availability

The data that support the findings of this study are available from the corresponding author upon reasonable request.
